# Exploring bycatch diversity of organisms in whole genome sequencing of Erebidae moths (Lepidoptera)

**DOI:** 10.1038/s41598-021-03327-3

**Published:** 2021-12-30

**Authors:** Hamid Reza Ghanavi, Victoria G. Twort, Anne Duplouy

**Affiliations:** 1 Department of Biology, Lund University, Ecology Building, Sölvegatan 37, 22362 Lund, Skåne Sweden; 2 The Finnish Museum of Natural History, Luomus, Zoology Unit, The University of Helsinki, Helsinki, Finland; 3 Insect Symbiosis Ecology and Evolution, Organismal and Evolutionary Biology Research Program, The University of Helsinki, Helsinki, Finland

**Keywords:** Biodiversity, Biodiversity, Entomology, Microbiology, Zoology, Genomics

## Abstract

Models estimate that up to 80% of all butterfly and moth species host vertically transmitted endosymbiotic microorganisms, which can affect the host fitness, metabolism, reproduction, population dynamics, and genetic diversity, among others. The supporting empirical data are however currently highly biased towards the generally more colourful butterflies, and include less information about moths. Additionally, studies of symbiotic partners of Lepidoptera predominantly focus on the common bacterium *Wolbachia pipientis*, while infections by other inherited microbial partners have more rarely been investigated. Here, we mine the whole genome sequence data of 47 species of Erebidae moths, with the aims to both inform on the diversity of symbionts potentially associated with this Lepidoptera group, and discuss the potential of metagenomic approaches to inform on host associated microbiome diversity. Based on the result of Kraken2 and MetaPhlAn2 analyses, we found clear evidence of the presence of *Wolbachia* in four species. Our result also suggests the presence of three other bacterial symbionts (*Burkholderia* spp., *Sodalis* spp. and *Arsenophonus* spp.) in three other moth species. Additionally, we recovered genomic material from bracovirus in about half of our samples. The detection of the latter, usually found in mutualistic association to braconid parasitoid wasps, may inform on host-parasite interactions that take place in the natural habitat of the Erebidae moths, suggesting either contamination with material from species of the host community network, or horizontal transfer of members of the microbiome between interacting species.

## Introduction

A growing scientific community now sees each organism as a community of interacting species rather than as an independent entity. Insects are no exception. They host a variety of microbial symbionts sitting both inside and outside their host cells. These microorganisms are at least as numerous as the number of host cells, and may constitute up to 10% of the host total mass^1^. The effects of symbionts on their insect hosts are potentially as diverse as their taxonomy, ranging from pathogenic to obligate mutualists, and all the intermediate possible relationships^2^. This diversity has recently attracted the growing interest of the scientific community, but gaps and biases remain. For example, in Lepidoptera, research in symbiosis has mostly focused on the most charismatic groups of colourful diurnal butterflies^3–5^ and on pest species to the human society^6–8^. In contrast, the rest of Lepidoptera (mainly moths), which encompass no less than 130,000 species^9^ (80% of all Lepidoptera), have rarely been screened for their associations with symbionts^10^.

High throughput sequencing technologies (HTS) now provide a relatively easy and cheap way to obtain large amounts of genetic data. These technologies used to generate genomic data are varied and broadly applicable to the widest range of organisms. Thereby, revolutionizing our accessibility to genomic resources and continually expanding and renewing the scope of the questions we can address within the natural sciences. For example, sequencing material from a particular study organism, either entirely or partially, may results in a mix of primary host specific DNA and DNA from other sources. These other sources can include ecto/endosymbionts, food, opportunistic parasites and pathogens, among others. Such genomic data opens up the genomic analyses towards broader targets, including towards investigating the diversity of symbionts that might be associated to particular targeted hosts.

Here, we mine the data produced from whole genome sequencing of 47 moth species from the hyper-diverse family Erebidae (24,000 species) to (1) explore the potential diversity of symbionts associated to this megadiverse Lepidoptera family; and (2) to evaluate the exploratory power of recovering information on natural host-symbiont associations from the low coverage genome sequencing approaches.

## Results

### Metagenomic analysis

**Figure 1:**
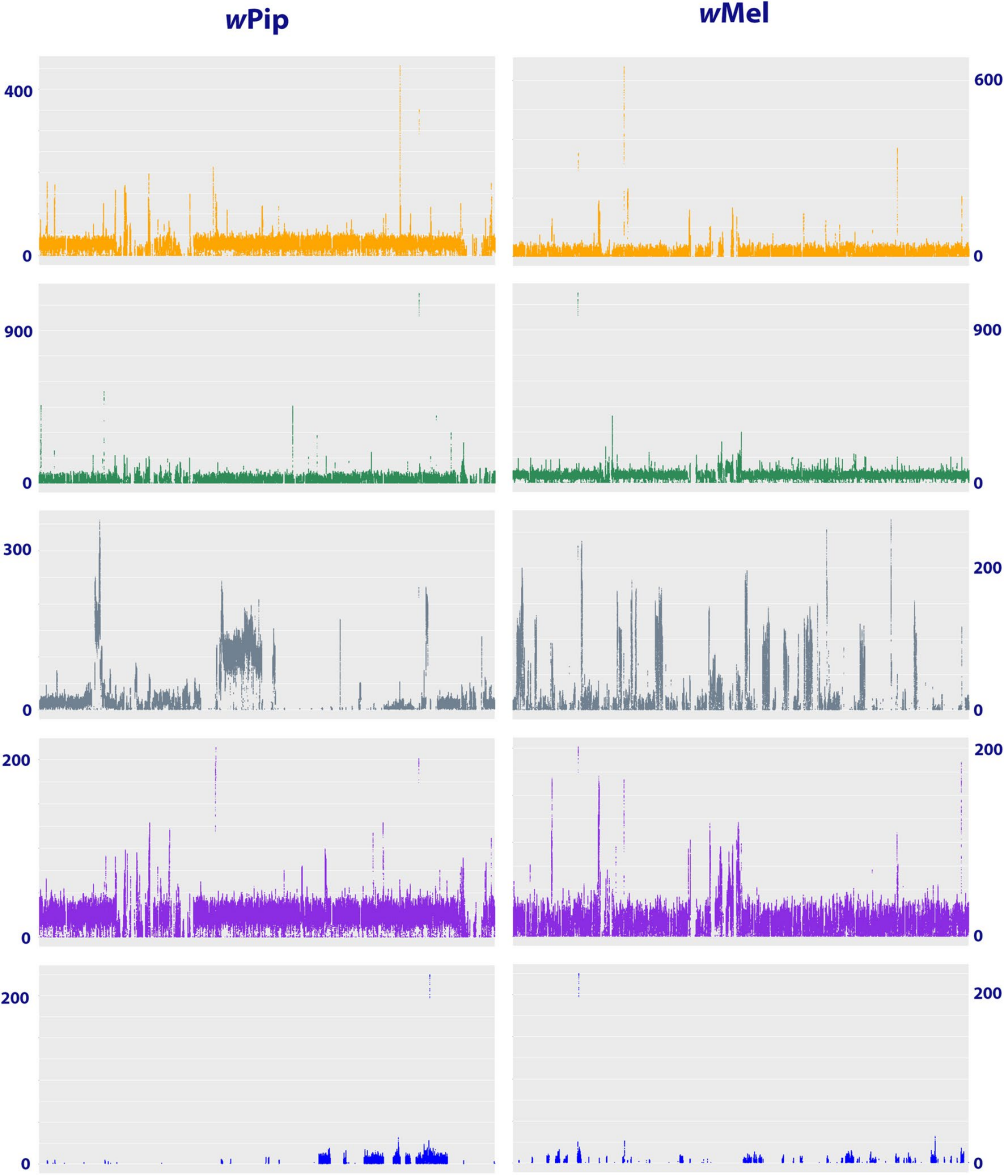
The mapped reads to *w*Mel and *w*Pip *Wolbachia* reference genomes. The coverage is shown on the vertical side of the figure. The top graphs (yellow) correspond to the sample RZ337 (*Oraesia excavata*), followed by RZ271 (*Idia aemula* in green), RZ248 (*Acantholipes circumdata* in grey), RZ42 (*Luceria striata* in purple) and at the bottom RZ13 (*Gonitis involuta* in blue).

We identified the species *Idia aemula*, *Luceria striata*, *Acantholipes circumdata* and *Oraesia excavata* (RZ271, RZ42, RZ248, and RZ337) as infected by *Wolbachia*, and *Wolbachia*-associated phage *WO* (Table 1), with between 66,978 and 208,044 of the reads identified as belonging to the symbiont. Additionally, the reads obtained from sample RZ13 (*Gonitis involuta*) was also found to include 954 *Wolbachia* reads, which is a higher number of reads than found for any of the clearly uninfected specimens, but is considerably less than any of the four clearly infected specimens listed above. The mapping of the reads to two known *Wolbachia* reference genomes (*w*Mel, GCF_000008025.1 and *w*Pip, GCF_000073005.1) show a relatively homogeneous coverage of the reference genomes (Fig. 1) with mean coverages between 10 and 40 times the reference genome (Table 2). In the case of RZ13 sample, even though the coverage seemed homogeneously scattered through the reference genome, the mean coverage was lower than 1x (Table 2).

**Table 1:** The number of reads classified as originating from the host and various microorganisms.

Ref	Code	Species	Country	# raw reads (Million)	Kraken2 Results											Metaphlan2 results
					Lepidoptera	Spiroplasma	Burkholderia	Sodalis	Arsenophonus	Rickettsia	Wolbachia	Wolbachia PhageWo	Ichnovirus	Bracovirus	Microsporidia	Wolbachia
1	MM00407	Scoliopteryx libatrix	FINLAND	38	2,266,973	–	289	–	–	–	–	–	–	–	–	–
2	RZ103	Rema costimacula	HONG KONG	22	907.037	–	–	**9.108**	**1.336**	–	–	–	–	266	–	–
3	RZ104	Saroba pustulifera	HONG KONG	21	1,649,430	–	–	–	–	–	–	–	–	–	–	–
4	RZ105	Hypocala deflorata	HONG KONG	48	3,231,681	–	59	–	–	–	–	–	–	–	–	–
5	RZ11	Erebus ephesperis	TAIWAN	106	8,550,697	64	298	–	–	–	–	–	–	**1.288**	–	–
6	RZ111	Platyjionia mediorufa	HONG KONG	26	995.385	–	–	**4.395**	**662**	–	–	–	–	–	–	–
7	RZ119	Schistorhynx argentistriga	HONG KONG	56	5,928,236	56	99	–	–	–	–	–	–	–	–	–
8	RZ13	Gonitis involuta	TANZANIA	17	1,254,304	–	83	–	–	–	**954**	–	317	102	–	**2.005**
9	RZ138	Micronoctua sp.	INDONESIA	107	11,736,010	100	126	50	–	–	–	–	–	–	–	–
10	RZ149	Hypopyra capensis	GHANA	53	4,808,838	–	107	–	–	–	–	–	–	–	–	–
11	RZ159	Rivula ochrea	GHANA	59	6,499,556	71	216	–	–	–	–	–	–	–	–	–
12	RZ18	Masca abactalis	INDONESIA	45	4,175,988	–	67	–	–	–	–	–	–	**1.381**	–	–
13	RZ180	Nodaria verticalis	GHANA	38	4,198,076	–	116	–	–	–	–	–	–	**1.731**	–	–
14	RZ21	Ophiusa coronata	MALAYSIA	42	2,653,381	–	76	–	–	–	–	–	–	–	–	–
15	RZ22	Azeta ceramina	COSTA RICA	55	4,926,573	64	85	–	–	–	–	–	–	–	–	–
16	RZ248	Acantholipes circumdata	UAE	28	3,085,527	–	–	–	–	–	**29.454**	**410**	–	–	–	**220.309**
17	RZ265	Rhesala imparata	HONG KONG	38	6,206,848	–	67	–	–	–	–	–	–	–	–	–
18	RZ268	Mecodina praecipua	HONG KONG	26	2,200,296	–	–	–	–	–	–	–	–	**790**	–	–
19	RZ271	Idia aemula	USA	52	6,897,287	–	112	–	–	–	**144.331**	**1.038**	–	**771**	–	**168.228**
20	RZ28	Brunia antica	HONG KONG	77	7,118,395	59	242	–	–	50	–	–	–	–	–	–
21	RZ3	Laspeyria flexula	HUNGARY	54	7,583,217	–	82	–	–	–	161	–	–	–	–	–
22	RZ30	Creatonotos transiens	HONG KONG	30	6,196,702	–	**1.995**	–	–	–	–	–	–	198	–	–
23	RZ313	Sypnoides fumosa	JAPAN	87	10,986,269	–	505	–	–	–	–	–	–	104	–	576
24	RZ331	Tinolius eburneigutta	THAILAND	33	3,112,193	–	85	–	–	–	–	–	–	159	–	–
25	RZ332	Anoba anguliplaga	GHANA	42	1,874,468	–	79	–	–	–	–	–	–	–	–	–
26	RZ336	Calyptra hokkaida	JAPAN	34	5,835,726	–	122	–	–	–	–	–	–	341	–	–
27	RZ337	Oraesia excavata	HONG KONG	38	3,147,679	–	65	–	–	–	**66.978**	**182**	–	581	–	**208.044**
28	RZ34	Nygmia plana	HONG KONG	19	1,026,248	–	–	–	–	–	–	–	–	–	–	–
29	RZ367	Hypena baltimoralis	USA	35	3,005,435	–	54	–	–	–	–	–	–	–	–	–
30	RZ389	Tamsia hieroglyphica	MALAYSIA	26	1,285,828	–	63	–	–	–	–	–	–	572	–	–
31	RZ39	Ericeia subcinerea	HONG KONG	80	7,549,078	–	133	–	–	–	65	–	–	–	–	–
32	RZ4	Colobochyla salicalis	HUNGARY	44	5,510,176	–	57	–	–	–	–	–	–	72	–	–
33	RZ40	Pangrapta bicornuta	HONG KONG	63	7,415,193	–	316	–	–	–	60	–	–	107	–	–
34	RZ404	Amerila astreus	MALAYSIA	45	4,649,942	–	72	–	–	–	–	–	–	130	–	–
35	RZ41	Metaemene atrigutta	HONG KONG	17	1,079,839	–	–	–	–	–	–	–	–	–	–	–
36	RZ42	Luceria striata	HONG KONG	27	3,065,608	–	75	–	–	–	**67.176**	**494**	–	–	–	**181.728**
37	RZ44	Asota heliconia	HONG KONG	40	3,763,381	–	54	–	–	–	–	–	–	**1.384**	–	–
38	RZ48	Sympis rufibasis	HONG KONG	52	5,491,409	–	208	–	–	–	–	–	–	–	–	–
39	RZ56	Phyllodes eyndhovii	TAIWAN	64	4,058,586	–	118	–	–	–	–	–	–	–	–	–
40	RZ57	Lygephila maxima	JAPAN	41	3,832,732	–	117	–	–	–	–	–	–	–	–	–
41	RZ58	Melipotis jucunda	USA	57	5,764,266	–	101	–	–	–	–	–	–	101	–	–
42	RZ59	Panopoda rufimargo	USA	42	4,715,473	–	237	–	–	–	–	–	–	–	–	–
43	RZ8	Syntomis phegea	HUNGARY	22	1,675,147	–	–	–	–	–	–	–	–	309	177	–
44	RZ89	Arctornis sp.	JAPAN	33	3,256,478	–	50	–	–	–	–	–	–	94	–	–
45	RZ9	Scolecocampa liburna	USA	52	3,132,323	–	137	–	–	–	–	–	–	–	–	–
46	RZ93	Epitausa dilina	COSTA RICA	41	4,202,446	–	–	–	–	–	–	–	–	–	–	–
47	RZ94	Alesua etialis	COSTA RICA	16	1,605,058	–	76	–	–	–	–	–	–	–	–	–

Values in bold highlight the values mentioned in the text, – represent samples with either zero or less than 50 reads classified.

**Table 2:** Samples screened for *Wolbachia* genomes.

Sample	Reference	Reads mapped	Covered bases	%	Mean coverage
RZ13	Wmel	4102	129,338	10.2	0.4
	Wpip	8712	214,530	14.5	0.7
RZ248	Wmel	95,663	453,378	35.8	9.3
	Wpip	212,353	774,465	52.2	18.5
RZ271	Wmel	413,711	1,124,837	88.7	39.4
	Wpip	196,202	908,917	61.3	15.6
RZ337	Wmel	127,490	808,811	63.8	11.4
	Wpip	267,514	1,157,313	78.1	20.8
RZ42	Wmel	110,993	831,256	65.6	10.8
	Wpip	212,555	1,139,840	76.9	18.2

The *w*Mel and *w*Pip are reference strains for the A- and B-*Wolbachia* supergroups, respectively. Covered bases column gives the length of the reference covered in bp. The column marked with % gives the percentage of the genome recovered.

Both Kraken2 and MetaPhlan2 analyses showed no to very few reads mapping to *Cardinium, Hamiltonella* or *Spiroplasma* bacteria, or to Microsporidian fungi, in any of the 47 datasets screened. In contrast, the specimens RZ103 and RZ111 (*Rema costimacula* and *Platyjionia mediorufa*) included considerably more reads from *Sodalis* bacteria (9108 and 4395, respectively), and from *Arsenophonus* bacteria (1336 and 662, respectively), than any other samples (maximum of 50 reads in any other sample). A closer look at the Kraken2 outputs from the latter two samples also revealed a possible infection with a *Plautia stali* symbiont (gammaproteobacteria; 3856 and 1914 reads, respectively), which was not detected in any of the other 45 samples. Additionally, the sample RZ30 (*Creatonotos transiens*) is the only one to show relatively high number of reads mapping to *Burkholderia* bacteria (N = 1995). Finally, we identified a considerable amount of reads from viruses of the *Polydnaviridae* family, and especially of the bracoviruses in three samples, *Erebus ephesperis*, *Masca abactalis* and *Asota heliconia* (RZ11, 1288 reads, RZ18, 1381 reads, and RZ44, 1384 reads). All other samples only include less than 750 reads, and more often no reads, for these viruses.

All details of the screen for the common symbionts can be found in Table 1, while all results from the Kraken2 and MetaPhlAn2 analyses can be found in the supplementary material and GitHub repository.

## Discussion

We confidently add four moth species (i.e., *Idia aemula*, *Luceria striata*, *Acantholipes circumdata* and *Oraesia excavata*) to the list of species hosting the intracellular alpha-proteobacterial symbiont *Wolbachia*^10^, confirmed through two screening methods (i.e., Kraken2 and MetaPhlAn). With only 4 out of 47 species (8%) found infected, this represents a lower infection rate than the current literature suggests (i.e., 16–79% of the studied lepidopteran groups infected with *Wolbachia*^11–16^). The general penetrance of *Wolbachia* however varies significantly among species, and is often low within infected populations^17^. Thus, with only one sample screened per species, our results are most likely underestimating the true infection rate within the Erebidae moths. Future broader screenings of different populations will provide more accurate natural infection rates for these species. Although microbial surveys in *Calyptra thalictra*^18^and *Lymantria dispar*^19,20^ did not highlight *Wolbachia* infections in these species, a recent screening of diverse moth species from Thailand, showed that two (22%; *Olepa *sp. and *Creatonotos transiens*) out of nine Erebidae species screened (ie. *Amata *sp*., Asota plana, Creatonotos transiens, Euplocia membliaria, Fodina contigua, Neochera inops, N. dominia, Olepa *sp*., Pareuchaetes pseudoinsulata*) were infected by the bacterial symbiont^21^.

Noticeably, we observe the presence of *Wolbachia* phage *WO* within the samples for which *Wolbachia* presence is strongly supported. The interaction of this bacteriophage with *Wolbachia* has been the focus of many evolutionary studies in recent years^22–26^. Previous research suggests that phage *WO* are associated with horizontal gene transfer in *Wolbachia,* and with genes that may affect the fitness of the bacterium^27,28^. These bacteriophages have been observed in practically all the studied genomes of *Wolbachia* up to date, with very few obligate mutualistic exceptions^22,29,30^. In the sample RZ13, species *Gonitis involuta*, a relatively high number of reads mapped to *Wolbachia* (1 K reads), although significantly lower than in the other four species (29–144 K reads), and no reads were mapped to phage-*WO*. In addition to the relatively lower sequencing depth compared to the other positive cases, few non-excluding hypotheses may explain such a pattern, these reads might originate from (1) contamination with other genetic material alien to our sample, (2) the integration of *Wolbachia* genomic material (partially or entirely) in the host genome, (3) random errors in the identification of the reads as *Wolbachia*, (4) low quality genomic material or (5) a combination of above-mentioned reasons. The overall screening results suggest that this sample was of low quality prior to sequencing. We however cannot rule out any of the other possibilities, and more studies are needed to fully confirm or reject the presence of *Wolbachia* in this species.

The two samples, *Rema costimacula* (RZ103) and *Platyjionia mediorufa* (RZ111), were of particular interests. Both the Kraken2 and the MetaPhlAn2 analyses suggest the presence of three gammaproteobacteria endosymbionts, namely *Sodalis*, *Arsenophonus and* ‘*Plautia stali-*symbiont’ in both samples. *Sodalis* has been characterized from different insects, including tsetse flies^31^, seal louse^32^, pigeon louse^33^, loose flies^34^, aphids^35^, seed bug^36^, weevils^37,38^, stinkbugs^39^, bees^40^, and ants^41^, among others. To our best knowledge however, this is the first time the three symbionts are found in Lepidoptera (Duplouy and Hornett^10^). This suggests that *Sodalis* bacteria might affect a more diverse group of organisms than is currently known. We are however cautious with the interpretation of this result, as the simple discovery of bacteria in the genomic data does not inform us about the nature of their interactions with the hosts. Whether *Sodalis* and the moth species share a symbiotic relationship, or not, will only be confirmed via experimentation and testing of the partnership through the host generations. Contamination of those two samples prior to DNA extraction is always possible. However, the sequenced host genetic material did not include significant amount of hemipteran DNA (or any other non-lepidopteran insect order), with comparable low numbers of reads (< 1500) mapped to hemipterans in all the sequenced genomes. This rules out DNA contamination by material from the previously confirmed hemipteran hosts of these three symbionts. It is shown that the female brown-winged green bug, *P. stali*, smears excrement over the egg surface during oviposition. The nymphs acquire the symbionts right after hatching by ingesting the excrements^42^. Therefore, a possible contamination source could be any contact with such excrement/egg clusters. Once again, studies of the symbionts in natural populations of these moth species are needed to fully resolve the true infection state of these species and the relationship with the bacteria.

The moth species *Creatonotos transiens* shows a potential partnership with proteobacteria *Burkholderia* sp. Recently, Boonsit and Wiwatanaratanabutr^21^ found *Wolbachia* in 75% of the *C. transiens* samples they screened for (N = 6/8). Their samples were collected from Thailand, while the *C. transiens* specimen we analysed in this study originated from Hong Kong. In Lepidoptera, *Burkholderia* are known from the microbiota associated with the moth *Lymantria dispar*^43^. However, similarly to the other symbionts presented above, these bacteria are also found in very diverse groups of organisms, from Amoebas to Orthoptera, from humans to plants^44–47^. In the bean bug, *Riptortus pedestris*, studies have suggested that the bacteria can benefit their host by providing resistance to pesticides^48^. Although never tested, the presence of such Proteobacteria in moths could similarly enhance the host ability to resist pesticides. If proven true, this could contribute to partially explaining the global success of many pest moth species despite the development of various targeted control strategies.

Six genomes included significantly high amounts of bracovirus reads, *Erebus ephesperis* (RZ11), *Masca abactalis* (RZ18), *Nodaria verticalis* (RZ180), *Mecodina praecipua* (RZ268), *Idia aemula* (RZ271) and *Asota heliconia* (RZ44). Bracoviruses are a known genus of mutualistic viruses with a complex life cycle. Integrated in the genome of a braconid parasitic wasp, the bracovirus is transcribed during oviposition in lepidopteran larvae^49^. The presence of this viral genetic material in adult moths might suggest an unsuccessful infection by the parasitoid, and the survival of the larvae carrying the parasitic viral particles. Another potential explanation includes the possibility for the viral DNA to be integrated into the lepidopteran genome, as it is usually found in its common Hymenoptera host. Only studies simultaneously investigating parasitism success rate and tissue tropism of the bracoviruses in the Lepidoptera and Hymenoptera hosts, will be able to inform on the nature of these interactions.

From a methodological point of view, the present study shows the successful exploratory approach to mine for potentially hidden associated microbial diversity in genomic data. Our study was performed on shallow genome short reads obtained using Illumina platform. The original purpose of this sequencing effort was to study the phylogenomics of the hosts species^50^, but a similar approach to the one we have taken here can be implemented to any publicly available genomic datasets. The popularity of genomic scale sequence data methods, such as Illumina short read approach, created a wide publicly open genomic resource for the research community to study questions that are not directly into the focus of the studies generating them. It is however important to also consider the limitations of such approaches. First, the quality and completeness of the reference datasets needed for programs like Kraken2 are bound to significantly affect the results. Second, incomplete and shallow genomes tend to present false negatives when mined for many symbionts. In addition, the origin of the DNA used for the genome sequencing will affect any conclusion on presence/absence or abundance of the symbionts detected and those undetected. In our study, all the used genomes came from DNA extracted from legs, therefore there is a methodical hard bias against gut fauna for example, however as shown in other studies some symbionts as *Wolbachia* are also found in the haemolymph of arthropods^51^. Third, this kind of exploratory analyses of genomic material does not inform about the nature of the interaction between the organisms found in the genomic mix. Furthermore, in the majority of cases, this method also does not inform on the origin of the organisms. This is especially important as sample contamination is a known problem since the appearance of the molecular sequencing techniques. Finally, this method is not suitable for quantification of the present organisms. Altogether, these limitations exemplify the exploratory nature of the approach we used in this study, and that we at best provide suspicion for diverse symbiotic infections in different Erebidae moth species, which presence and importance will only be fully confirmed via direct screening, and ecological and evolutionary studies of natural populations.

## Conclusion

As we expected, our method detects various symbiotic partners in several Erebidae moth species, including *Wolbachia* and the bacteriophage *WO* in four species, *Burkholderia* in one other species, and *Sodalis* and *Arsenophonus* simultaneously in two species*.* Although symbiotic associations of Lepidoptera with *Wolbachia* is likely, similar long-term associations between the three other symbionts and the Lepidoptera have yet to be described. Similarly, we detect DNA material from bracoviruses that are currently only described as mutualistic symbionts of Hymenoptera. The true nature of these associations requires further experimental investigation. The detection of bracovirus DNA could for example suggest ecological interactions between moths and parasitoids, and the ability of the formers to naturally resist parasitoid attack strategies. Altogether our study presents a method and produces material supporting testable hypotheses about the diversity and nature of symbiotic interactions in those particular Lepidoptera species. With the availability of open access metagenomics databases, this field promises extensive and exciting opportunities to explore potentially hidden symbiotic diversity.

## Material and methods

### Genome data

We used the data produced from the whole genome sequencing project of 47 Erebidae species (see^50^). The sampling information is shown in Table 1. This selection includes genomes representing the main described subfamilies and major lineages within the Erebidae family. The DNA was extracted from one or two legs of the selected samples. Extractions took place in 2000 s/over a decade ago, for the purpose of another study (see^52^). It is important to keep in mind that the genome sequencing approach generating this dataset is not optimized to recover the symbiont diversity of these organisms, therefore the diversity is likely to be systematically underestimated.

### Metagenomic analysis

The raw reads were quality checked with FASTQC v0.11.8^53^. Reads containing ambiguous bases were removed from the dataset using Prinseq 0.20.4^54^. Reads were cleaned to remove low quality bases from the beginning (LEADING: 3) and end (TRAILING: 3) and reads less than 30 bp in length. The evaluation of read quality with a sliding window approach was done in Trimmomatic 0.38^55^. Quality was measured for sliding windows of 4 bp and had to be greater than PHRED 25 on average. Cleaned reads were assigned taxonomic labels with Kraken2^56^ and MetaPhlAn 2.0^57^. Kraken2 was run using a custom database, which contained the standard kraken database, the refseq viral, bacteria and plasmid databases and all available Lepidoptera genomes from genbank (Supplementary Table 1 contains a full list of taxa included), confidence threshold of 0.05, and a mpa style output. MetaPhIAn was run using the analysis type rel_ab_w_read_stats, which provides the relative abundance and an estimate of read numbers originating from each clade. We visually screened the result for each sample, focusing on seven genera of vertically transmitted bacterial symbionts (i.e., *Arsenophonus* sp., *Cardinium* sp., *Hamiltonella* sp., *Rickettsia* sp., *Sodalis* sp., *Spiroplasma* sp. and *Wolbachia* sp.), one group of fungal symbionts (Microsporidia), and three types of viral symbionts (i.e., *Wolbachia*-phage *WO*, ichnovirus and bracovirus). This represents a non-exhaustive list of the maternally inherited symbionts found in diverse insect hosts, but covers all of those that have already been characterized within Lepidoptera^10^. We also checked on the presence of the gut bacteria *Burkholderia* sp., which are known to confer pesticide resistance to their host in the pest bean bug *Riportus pedestris* (e.g., ‘can degrade an organophosphate pesticide, fenitrothion’)^58^.

To discriminate between true and false positives a mapping analysis was carried out. For *Wolbachia* positive samples (list), cleaned reads were mapped to both the *w*Mel (GCF_000008025.1) and *w*Pip (GCF_000073005.1) genomes uses bowtie2 v2.4.1^59^ (sensitive local option). The resulting sam files were converted to sorted bam files with samtools v1.10^60^. Coverage information was obtained using samtools depth, and the resulting graphs plotted with ggplot package^61^ in R.

## Supplementary Information

Supplementary Information.

## Data Availability

The genome data used in this study are deposited in the NCBI SRA under BioProject PRJNA702831. All data in the supplementary material, the tables and the results can be found and downloaded from the GitHub repository: github.com/Hamidhrg/ErebidSymbionts.
